# Associations between subjective well-being and subcortical brain volumes

**DOI:** 10.1038/s41598-017-07120-z

**Published:** 2017-07-31

**Authors:** D. Van ‘t Ent, A. den Braber, B. M. L. Baselmans, R. M. Brouwer, C. V. Dolan, H. E. Hulshoff Pol, E. J. C. de Geus, M. Bartels

**Affiliations:** 1 Department of Biological Psychology, VU University, Amsterdam, The Netherlands; 2 EMGO+ Institute for Health and Care Research, VU University Medical Center, Amsterdam, The Netherlands; 3 Amsterdam Neuroscience, Amsterdam, The Netherlands; 4 Alzheimer Center and Department of Neurology, VU University Medical Center, Amsterdam, The Netherlands; 5 Brain Center Rudolf Magnus, Department of Psychiatry, University Medical Center Utrecht, Utrecht, The Netherlands

## Abstract

To study the underpinnings of individual differences in subjective well-being (SWB), we tested for associations of SWB with subcortical brain volumes in a dataset of 724 twins and siblings. For significant SWB-brain associations we probed for causal pathways using Mendelian Randomization (MR) and estimated genetic and environmental contributions from twin modeling. Another independent measure of genetic correlation was obtained from linkage disequilibrium (LD) score regression on published genome-wide association summary statistics. Our results indicated associations of SWB with hippocampal volumes but not with volumes of the basal ganglia, thalamus, amygdala, or nucleus accumbens. The SWB-hippocampus relations were nonlinear and characterized by lower SWB in subjects with relatively smaller hippocampal volumes compared to subjects with medium and higher hippocampal volumes. MR provided no evidence for an SWB to hippocampal volume or hippocampal volume to SWB pathway. This was in line with twin modeling and LD-score regression results which indicated non-significant genetic correlations. We conclude that low SWB is associated with smaller hippocampal volume, but that genes are not very important in this relationship. Instead other etiological factors, such as exposure to stress and stress hormones, may exert detrimental effects on SWB and the hippocampus to bring about the observed association.

## Introduction

Feelings of happiness are essential to the overall mental health. Happy people tend to function better in life, are typically more productive and socially engaged and tend to have higher incomes^1, 2^. Ryan and Deci^3^ pointed out that happy people or, more generally, people characterized by a high degree of subjective well-being tend to have attribution styles that are more self-enhancing and more enabling than those low in well-being. This suggests that positive emotions can lead to positive cognitions, which, in turn, contribute to further positive emotions.

A number of twin studies have established the contribution of genetic factors to variation in behavioral traits of “positive psychology”, such as happiness, satisfaction with life, and quality of life, either independently or under the umbrella of subjective well-being (SWB). Heritability estimates are around 40%^4, 5^. Furthermore, molecular genetic influences^6^ and the first genetic variants that explain differences in subjective well-being^7^ have been reported recently. The genetic contribution to variation in SWB raises the question of its neurobiological foundation. Initial work primarily pointed to the involvement of the frontal cortex. First, Urry *et al*.^8^, using electroencephalography (EEG), found greater left than right prefrontal cortex activation in subjects who reported higher well-being. This finding received much attention as it provided an experimental link between eudaimonic well-being and an approach-oriented behavioral style, which was related to the left hemisphere in earlier studies^9^. Following up on this work, physiological activity in the frontal left hemisphere has been associated with several traits of positive psychology, such as quality of life^10, 11^, positive attitude, high self-esteem and optimism^12^. Involvement of the anterior brain in general (i.e., not limited to the left hemisphere) has also been reported for eudaimonic well-being^13, 14^, happiness^15^ and optimism^16^. However, two recent structural MRI studies found no support for a role of the frontal brain, but observed an association of insular grey matter volume with general eudaimonic well-being^17^, and right precuneus volume with subjective happiness^18^.

In addition to the cortex, a role of subcortical structures in relation to well-being may be anticipated. In particular the hippocampus and amygdala are assumed to be involved in the regulation of emotion and cognition^19^. Furthermore, the nuclei of the basal ganglia (caudate, putamen, pallidum), accumbens and the thalamus are key components in regulating reward processing in the brain^20^. The thalamus is also involved in emotion regulation through its connections with the limbic system^21^. To date, however, little work has been devoted to identifying the subcortical underpinnings of well-being. We found one structural MRI study that reported a positive correlation with gray matter volume of the left thalamus/left pulvinar for dispositional optimism^21^. In addition, one resting-state fMRI study reported increased homogeneity of functional activation in the right putamen and left thalamus in individuals who scored low on subjective happiness^15^. One task fMRI study found more sustained striatal activity in subjects that reported greater eudaimonic well-being^13^.

However, most of the available evidence implicates the amygdala and the hippocampus, or closely associated medial temporal lobe regions. With regard to the amygdala, functional studies reported a positive correlation between amygdala activation and trait happiness^22^ and between amygdala activation and imagination of positive relative to negative future events^16^. Negative correlation of amygdala activition with a purpose of life scale has also been noted^14^. One whole brain VBM study on dispositional optimism^21^ and one on global life satisfaction^23^ reported positive correlations with grey matter volume of the medial temporal lobes including the parahippocampal gyrus. In addition, two structural MRI studies focused specifically on the hippocampus and found that smaller hippocampal volume was related to lower self-esteem^24, 25^. Interestingly, medial temporal lobe involvement is consistent with the hypothesis that the neural substrates of reduced well-being are similar to those of depression and stress. Neuroimaging research on anxiety/depression and stress related disorders has yielded ample evidence for structural impairment of the hippocampus^26, 27^ and also, albeit less consistently, for impairment of the amygdala^28, 29^.

In this study we aimed to further our understanding of the subcortical underpinnings of SWB in a large dataset of 724 monozygotic (MZ) twins, dizygotic (DZ) twins and siblings with both structural MRI of the brain and measured subjective well-being. In this largest study to date on the neurobiology of well-being, we tested for associations with left and right hemisphere volumes of the basal ganglia, thalamus, hippocampus, amygdala and nucleus accumbens. To investigate significant SWB-brain associations we applied Mendelian Randomization (MR)^30^ to test for the alternative possibilities that SWB variation attributable to genes influences brain volume(s) or conversely, that brain volume variation attributable to genes influences SWB. To address these questions, we used polygenic risk scores for SWB and brain volume(s), that were computed for each individual from summary statistics provided by recently published genome-wide association (GWA) meta-analyses on SWB^7^ and volumes of subcortical brain structures^31^. In addition, to test for relative contributions of genetic and environmental factors to SWB-brain associations we applied bivariate twin modeling on our dataset. Variation in brain structures has been found to be heritable in twin-studies^32, 33^. Finally, we obtained an estimate of the genetic correlation between the brain volume(s) and SWB by comparing the published GWA summary statistics for brain volume^31^ and SWB^7^ using bivariate LD score regression^34, 35^.

## Results

### Phenotypic associations

**Figure 1:**
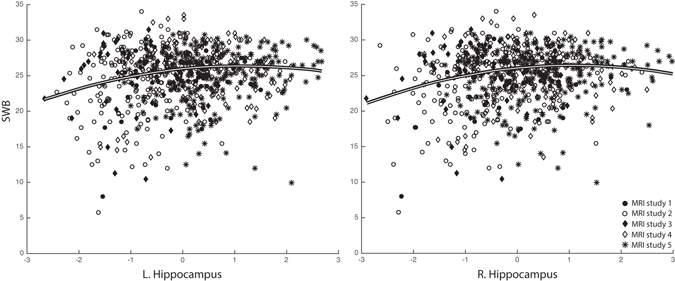
The composite subjective well-being scores (SWB) plotted against, Z-transformed, hippocampal volumes, for each of the 5 included studies. Fit lines indicate non-linear relations, between SWB and left and right hippocampal volume, as estimated using mixed model analysis across all available data points.

Linear mixed model results of the tests for association between SWB and subcortical volumes are listed in Table 1. Only volumes of the left and right hippocampal regions were found to be significantly associated with SWB. The association included a contribution of both the linear predictor term (left hemisphere: p = 0.001; right hemisphere: p < 0.001) and quadratic term (left hemisphere: p = 0.008; right hemisphere: p = 0.003). The curvilinear relations, visualized in Fig. 1, indicate that smaller hippocampal values are associated with reduced SWB while medium to larger hippocampal values are associated with comparable levels of SWB. Computed mean SWB values for low (Z score ≤ −0.5), medium (−0.5 < Z score < 0.5) and high (Z score ≥ 0.5) hippocampal volumes, respectively, were 24.1 ± 4.9, 25.3 ± 3.7 and 25.6 ± 3.4. We found no significant relations between SWB and the other fixed variables, i.e., ICV, sex, age at MRI and no significant effects for the factor MRI study.

**Table 1:** Statistical results of mixed model tests (Est.: standardized regression point estimate; S.E.: standard error; df: denominator degrees of freedom; F: F-value; sig.: p-value) for linear (column Linear) and quadratic (column Quadratic) associations between subcortical brain volumes and SWB.

Brain region	Linear					Quadratic				
	Est.	S.E.	df	F	sig.	Est.	S.E.	df	F	sig.
L. Caudate	0.284	0.216	600.1	1.735	0.188	−0.055	0.112	594.6	0.242	0.623
R. Caudate	0.343	0.202	604.9	2.878	0.090	−0.051	0.107	585.3	0.231	0.631
L. Putamen	−0.041	0.245	579.3	0.027	0.869	0.103	0.117	589.9	0.768	0.381
R. Putamen	−0.042	0.243	576.0	0.029	0.864	0.132	0.121	623.4	1.198	0.274
L. Pallidum	0.016	0.222	673.1	0.005	0.943	−0.027	0.115	662.1	0.057	0.812
R. Pallidum	0.211	0.196	706.0	1.155	0.283	0.183	0.105	709.7	3.049	0.081
L. Accumbens	−0.122	0.242	712.6	0.253	0.615	0.046	0.111	703.5	0.175	0.676
R. Accumbens	0.277	0.274	697.7	1.022	0.312	−0.073	0.121	688.7	0.369	0.544
L. Thalamus	0.341	0.293	657.6	1.354	0.245	−0.118	0.128	591.5	0.852	0.356
R. Thalamus	0.515	0.262	651.3	3.849	0.050	−0.121	0.115	627.6	1.102	0.294
L. Hippocampus	0.746	0.230	658.0	10.513	0.001*	−0.324	0.122	690.2	6.979	0.008
R. Hippocampus	0.725	0.206	668.2	12.322	0.000*	−0.335	0.112	705.7	8.918	0.003*
L. Amygdala	−0.140	0.247	709.9	0.321	0.571	0.061	0.102	688.3	0.353	0.552
R. Amygdala	0.403	0.225	686.4	3.192	0.074	−0.122	0.111	684.6	1.215	0.271

*Statistically significant at the 0.0036 level

### Post-hoc assessments

To examine the observed relations between SWB and hippocampus volumes in more detail, we performed a number of post-hoc tests. The results of these assessments, which can be classified as tests regarding the dependent variables of interest, and tests of the independent variables of interest and covariate control, are summarised in Table 2.

**Table 2:** Statistical results of different post-hoc modifications to the association tests with left or right hippocampal volume (Test type: type of modification; Modification: applied change; Brain region: L. Hipp./R.hipp. = left or right hippocampus volume as variable of interest; Linear: linear term; Quadratic: quadric term, with Est.: standardized regression point estimate; S.E.: standard error; df: denominator degrees of freedom; F: F-value; sig.: p-value.

Test type	Modification	Brain region	Linear					Quadratic				
			Est.	S.E.	df	F	sig.	Est.	S.E.	df	F	sig.
Dependent variable	Use SAT score instead of SWB	L. Hipp.	0.856	0.262	654.2	10.693	0.001	−0.350	0.140	688.0	6.296	0.012
		R. Hipp.	0.626	0.235	662.9	7.080	0.008	−0.396	0.128	703.1	9.606	0.002
	Use HAP score instead of SWB	L. Hipp.	0.522	0.237	590.3	4.855	0.028	−0.150	0.125	621.3	1.435	0.231
		R. Hipp.	0.627	0.214	594.6	8.571	0.004	−0.116	0.116	631.8	0.987	0.321
Independent variables of interest	Drop quadratic volume term	L. Hipp.	0.640	0.227	661.0	7.937	0.005					
		R. Hipp.	0.633	0.205	673.6	9.536	0.002					
	No Z-transform of the volume terms	L. Hipp.	0.013	0.004	687.9	8.536	0.004	−0.001	0.001	690.2	6.979	0.008
		R. Hipp.	0.016	0.005	703.3	10.660	0.001	−0.002	0.001	705.7	8.918	0.003
Covariate control	Add Age at SWB	L. Hipp.	0.759	0.231	658.8	10.742	0.001	−0.327	0.124	683.9	6.949	0.009
		R. Hipp.	0.747	0.207	668.2	12.959	0.000	−0.347	0.113	702.2	9.435	0.002
	Add Psychiatric symptom score	L. Hipp.	0.648	0.220	638.8	8.660	0.003	−0.285	0.118	677.9	5.862	0.016
		R. Hipp.	0.606	0.198	644.6	9.398	0.002	−0.244	0.108	692.9	5.099	0.024

### Test type: dependent variables of interest

In our primary analyses we used a subjective well-being (SWB) measure, i.e., the mean value across scores on a satisfaction with life (SAT) scale and a subjective happiness (HAP) scale. In a first post-hoc step, we tested the associations with either SAT or HAP scores alone. Mixed model results in Table 2 (Modification: Use SAT score instead of SWB; Modification: Use HAP score instead of SWB) indicate statistical significance values for the volume terms that were similar to our original findings for the composite SWB score, except that there was less evidence for a contribution of the quadratic terms when using HAP scores.

### Test type: independent variable of interest

In our association tests, we included both linear and quadratic volume terms. Firstly, we examined whether the non-linear term could be dropped to render the relationship linear. To this end, we excluded the quadratic terms, and refitted the mixed model associations between SWB and left and right hippocampal volumes. Compared to the original test statistics (F-value and sig.: p-value) for the linear term in Table 1, the post-hoc fit results listed in Table 2 (row Modification: Drop quadratic volume term) showed slightly reduced statistical significance, indicating that adding a quadratic term improves the fit to the data.

We tested for associations after applying Z-score transformation of the subcortical volumes. In an additional post-hoc test, we examined the effect of Z-tranformation by refitting the model with the original, unstandardized, volumes. The results in Table 2 (row Modification: No Z-transform of the volume terms) indicate a slight effect on the statistical significance values of the linear terms, but no effect on the quadratic terms.

### Test type: covariate control

In our subjects, the age at which hippocampal volumes were assessed (Age_MRI_) differed from the age at which well-being was measured (Age_SWB_). Given hippocampal volume changes across ages^36^ as well as possible changes in SWB^37, 38^, we performed an additional association test in which we included both Age_MRI_ and Age_SWB_ as covariates. This adjustment actually lead to slightly increased statistical significance values compared to the originally observed associations, in which we controlled for Age_MRI_ only (Table 2, Modification: Add Age at SWB).

Finally, we note that we used a sample composed of participants with MRI data collected in 5 separate studies that differed not only in sample size, but also with respect to participant selection. For example with regard to age of the subjects, MRI studies 1 and 5 include adolescents around 15 and 9 years old respectively, while studies 2, 3 and 4 include only adult subjects. Another difference is that for studies 1, 2 and 3, additional selection was applied based on high or low scores on symptoms for ADHD, OCD and depression, respectively. The high compared to low scoring subjects in these studies showed significantly different scores for SWB (see Table 3).

**Table 3:** SWB scores (N: number of subjects; Mean: group mean SWB score and SD: standard deviation) in subjects that score either high (column Symptom score: High) or low (column Symptom score: Low) on symptoms for ADHD (Study 1), OCD (Study 2) or depression (Study 3). Column High vs.

Study	Symptom score						High vs. Low		
	High			Low					
	N	Mean	SD	N	Mean	SD	df	F	sig.
1	23	22.6	4.3	35	25.7	2.6	56	13.5	0.001
2	75	22.0	4.9	167	25.6	3.6	240	40.7	<0.001
3	23	21.5	4.7	35	27.2	2.2	56	38.8	<0.001

Low shows the results of the statistical comparison of SWB scores in subjects with high vs. low symptom scores (df: degrees of freedom; F: F value; sig.: p-value).

These different sample characteristics may have introduced differences in the observed SWB-hippocampus volume associations. We tested for this post-hoc, firstly by creating an additional covariate regressor that coded for psychiatric symptom score (0: subjects with low symptom score; 1: phenotypically healthy/unselected subjects; 2: subjects with high symptom score). Including this variable as an additional regressor marginally reduced the statistical significance values for our associations (Table 2, Modification: Add Psychiatric symptom score), indicating a limited effect of symptom selection.

Secondly, we repeated our mixed model analyses to specifically test for differences in the effects over studies (i.e., by including study by linear and study by quadratic interaction terms). In testing the interactions, we conducted omnibus test for the 4 interactions with an alpha of 0.05/4 = 0.0125. Following a significant omnibus test of interaction, we inspected the specific contrasts with study 5 as the reference. We adopt a more liberal alpha of 0.05 in exploring the individual contrasts.

The omnibus test indicated no interactions for the linear volume terms (left hippocampus: F(4, 678.5) = 0.8, p = 0.541; right hippocampus: F(4, 666.2) = 1.1, p = 0.339), but did point to between study differences for the quadratic terms (left hippocampus: F(4, 650.0) = 3.2, p = 0.012; right hippocampus: F(4, 660.6) = 4.5, p = 0.001). With study 5 as the reference study, we found that the quadratic term associated with the left hippocampus differed in studies 1 (t(687.7) = −2.4, p = 0.015) and 4 (t(686.8) = −3.0, p = 0.003) and that the quadratic term associated with the right hippocampus differed in studies 1 (t(551.8) = −3.9, p < 0.001), 2 (t(698.1) = −2.3, p = 0.025) and 4 (t(694.7) = −2.1, p = 0.041).

In Table 4, we also show estimated linear and quadratic hippocampal volume predictors for SWB from mixed models conducted separately in each MRI study sample. The bottom row of Table 4 lists pooled meta-analysis results obtained by combining the model fit results for each individual MRI study using a weighted least squares (WLS) method^39^. In line with our findings based on mega-analysis across all available data, the meta-analyses confirm the presence of associations between SWB and left and right hippocampal volumes (given an alpha of 0.05/4 tests = 0.0125).

**Table 4:** Point estimates (column: Estimate) of the regression coefficients and associated test statistics (SE: standard errors; df: degrees of freedom; sig.: p-value), for the linear (column: Linear) and quadratic (column: Quadratic) volume predictors computed separately per study.

Study	Left Hippocampus								Right Hippocampus							
	Linear				Quadratic				Linear				Quadratic			
	Estimate	SE	df	sig.	Estimate	SE	df	sig.	Estimate	SE	df	sig.	Estimate	SE	df	sig.
1	0.135	0.275	43.5	0.626	−0.288	0.157	40.2	0.075	0.438	0.201	48.5	0.034	−0.419	0.099	49.1	<0.001
2	0.252	0.081	207.7	0.002	−0.067	0.049	229.6	0.166	0.258	0.082	194.7	0.002	−0.096	0.049	231.0	0.051
3	−0.006	0.195	44.7	0.977	−0.076	0.067	32.2	0.268	−0.193	0.228	43.8	0.401	−0.194	0.092	44.8	0.041
4	−0.037	0.107	109.2	0.731	−0.229	0.068	116.6	0.001	−0.016	0.111	107.1	0.884	−0.116	0.070	123.5	0.102
5	0.058	0.080	219.2	0.467	0.027	0.049	226.0	0.587	0.055	0.080	225.9	0.494	0.046	0.048	225.7	0.348
Pooled	0.105	0.048	4.0	0.027	−0.073	0.028	4.0	0.009*	0.122	0.048	4.0	0.011*	−0.086	0.028	4.0	0.002*

The bottom row shows the pooled meta-analysis results obtained by combining the model fit results for each individual MRI study using weighted least squares (WLS).

*Statistically significant at the 0.0125 level.

### Mendelian Randomisation

We subsequently determined whether the observed relation between SWB and hippocampal volumes could be interpreted causally. In the test for the causal influence of SWB on hippocampal volume, we confirmed that the polygenic scores for SWB predicted SWB in our subjects (d.f. = (1,373.9); F = 5.6; p = 0.019). However, the 2 stage least squares (2SLS) Mendelian Randomisation test results indicated no significant relations between hippocampal volumes and the polygenic risk scores for SWB (see Table 5: top row).

**Table 5:** Results (df: degrees of freedom; F: F-value, sig.: p-value) of 2 stage least square (2SLS) tests for a causal influence of SWB on hippocampal volume (top row) or conversely, a causal influence of hippocampal volume on SWB (bottom row), performed separately for the left and right hippocampus.

Causal Direction	Left Hippocampus			Right Hippocampus		
	df	F	sig.	df	F	sig.
SWB on Hippocampus	398.3	0.04	0.837	396.3	0.3	0.575
Hippocampus on SWB	345.3	2.2	0.138	356.4	1.8	0.185

Conversely, in the test for the causal influence of hippocampal volume on SWB, we again confirmed that the polygenic scores for hippocampal volume predicted left and right hippocampal volumes in our subjects (left hippocampus: d.f. = (1,392.7); F = 10.9; p = 0.001; right hippocampus: d.f. = (1,388.5); F = 9.4; p = 0.002). However, the 2SLS Mendelian Randomisation test also did not support causality in this direction (Table 5: bottom row). In both directional tests there was no evidence for significant interactions with strata of the exposure variable in the second step of the 2SLS procedure.

### Twin modeling

We fitted the saturated model to obtained estimates of the 10 × 10 covariance matrices in the MZ and DZ twins, with sex, age and MRI study as fixed covariates and subsequently applied the ADE model (as described in the Materials and Methods section). For both the hippocampal volume measures and SWB, twice the DZ correlations were slightly smaller than the MZ correlations (Table 6), which suggests a possible role of dominance genetic effects. However, in the ADE model, the parameters associated with the dominance genetic covariance matrix (denoted Σ_D_, see Materials and Methods) were small. We tested these by dropping them from the model (i.e., setting Σ_D_ to equal zero) and the results suggested that Σ_D_ can be omitted (Chi^2^(15) = 9.27, p = 0.862). Estimated heritabilities (percentage of variance attributable to additive genetic effects) by the resulting AE model are listed in the right column of Table 6. The genetic correlation between left and right hippocampus volumes (volumes squared) was 0.953 (0.871). Genetic correlations between SWB and left and right hippocampal volume were low: 0.078 (left), 0.044 (left squared), 0.069 (right) and 0.071 (right squared). The omnibus test of these correlations indicated that they did not differ from zero (Chi^2^(4) = 1.41, p = 0.842). The environmental correlations between left and right hippocampus volumes (volumes squared) equaled 0.296 (0.357). The environmental correlations between SWB and left and right hippocampal volume were 0.167 (left), −0.216 (left squared), 0.189 (right) and −0.313 (right squared). The omnibus test of these correlations indicate that they did differ from zero, given an alpha of 0.01 (Chi^2^(4) = 29.15, p < 0.001). The results indicate that the relationship between SWB and hippocampal volume is due to common unshared environmental effects.

**Table 6:** MZ and DZ twin correlations for hippocampal volumes, squared hippocampal volumes and the SWB score and associated heritabilities (column h^2^) as computed using an AE model.

Trait	Twin pair zygosity		h^2^
	MZ	DZ	
L. Hippocampus	0.750	0.285	0.762
L. Hippocampus^2^	0.659	0.277	0.636
R. Hippocampus	0.774	0.312	0.787
R. Hippocampus^2^	0.656	0.328	0.637
SWB	0.438	0.163	0.444

### Genetic Correlation from published GWAS

The absence of genetic associations between SWB scores and hippocampus volumes in our sample was confirmed by the fact that we did not find a significant genetic correlation (rg) when comparing the GWAS summary statistics reported for SWB (with median test statistics λ_GC_ = 1.206)^7^ and hippocampus volume (median test statistics λ_GC_ = 1.009)^31^: *rg* = −0.071 ± 0.1021, Z = −0.695, p = 0.487.

## Discussion

This study is the first to reveal (non-linear) relationships between individual differences in subjective well-being and volumes of the left and right hippocampus. In the literature, few studies investigated the neural structural correlates of well-being related traits. Most probed the brain for linear relations using voxel-based morphometry (VBM). Two of these studies reported associations with parts of the medial temporal lobes. One found a positive correlation for dispositional optimism with gray matter volume of the left thalamus/left pulvinar, extending to the left parahippocampal gyrus^21^. The other focused on global life satisfaction and reported a positive correlation with grey matter volume of the right parahippocampal gyrus, and negative correlations with volumes of the left precuneus and left ventromedial prefrontal cortex^23^. Two studies specifically focused on the hippocampus. Pruessner and colleagues used self-esteem as the behavioral variable and found, in line with our findings, that lower self-esteem was associated with smaller hippocampal volumes^24^. Later Kubarych *et al*.^25^ replicated this relation for self-esteem, but not for an additional measure of psychological well-being, notwithstanding the strong correlation between self-esteem and well-being.

The present study supports the association between subjective well-being and hippocampal volume. The non-linearity of this association implies that SWB is lower in subjects with a relatively smaller hippocampus, but similar in subjects with average to larger hippocampal size. Although testing for non-linear underlying functions is common in studies that investigate age effects on brain structure^40^, non-linearity has not been generally considered in studies on the relation between brain and behavior, or in fact any other factor that may relate to the brain structure. Although we are the first to show this for well-being, non-linear relationships due to stronger associations on the negative as compared to the positive end of the brain-behavior spectrum are in fact quite plausible. An obvious example is abnormal loss of behavioral function that results from brain structure impairment due to disease, but similar asymmetries have been reported for associations within the normal range of volumetric brain variation^41^.

We found no associations with volumes of the other subcortical structures tested, including the nuclei of the basal ganglia, thalamus, amygdala, and nucleus accumbens. Especially for the amygdala, associations with SWB are expected. Like with the hippocampus, volumetric changes of the amygdala have been implicated in depression^28^ and stress^29^, although not consistently^27^. In addition, several functional studies reported an association of amygdala activation with well-being^14, 16, 22^. However, to our knowledge, no structural studies support an association. In addition to the amygdala, associations may be expected with nuclei of the basal ganglia, accumbens, and the thalamus, as these structures are key components in regulating reward processing in the brain^20^, and in emotion regulation^21^. However, consistent with our results, there is also little support for these expectations. As noted earlier, one whole brain VBM study reported a positive correlation with gray matter volume of the left thalamus/left pulvinar for dispositional optimism^21^. In addition, one resting-state fMRI study found increased regional homogeneity of right putamen and left thalamus activity in unhappy individuals^15^.

Assessment of causal directions for the observed SWB-hippocampal volume relation by means of Mendelian Randomization (MR) yielded no evidence for a pathway from genes to SWB to hippocampal volume or oppositely, a pathway from genes to hippocampal volume to SWB. Given that our sample is relatively small for an MR approach^42^ these null findings may reflect a lack of statistical power. Notwithstanding, the absence of causal directions induced by genes is in line with the results of twin modeling on our sample and comparison of GWAS results for SWB^7^ and hippocampus volume^31^, by means of LD score regression, that both indicated no significant genetic correlations between SWB and hippocampal volume. The present observations are also consistent with twin modeling results of Kubarych *et al*.^25^, that indicated largely different genetic underpinnings of reported self-esteem and psychological well-being on one hand, and measured left and right hippocampal volumes on the other hand.

The lack of evidence for genetic influences suggests a role of other etiological factors. Our twin modeling results, which did indicate a significant contribution of unshared environmental effects, also support this. In this regard, factors associated with stress burden have been frequently mentioned^43–46^. The relation between reduced SWB and structural impairment of the hippocampus may then be explained by the hypothesis that reduced SWB is associated with stress and increased levels of stress hormones, such as cortisol^47^, which harm the hippocampus^48^. Besides neurotoxicity due to stress hormones, stress induced reductions of neurotrophic factors, especially Brain Derived Neurotrophic Factor (BDNF)^49^, and impaired neurogenesis^50^ may play a role. The premise of stress-induced harm to the hippocampus does not necessarily imply causal directions from reduced SWB and stress to final hippocampal damage. Hippocampal impairment may also be a risk factor for reduced SWB. For example, adverse stress effects due to early life etiological factors such as lack of parental care^43^ and childhood stress^44^ may result in impaired hippocampal development, which renders one more vulnerable to reduced SWB in later life. It is also likely that vulnerability, due to early life events, and adverse effects of stress and reduced SWB in later life both play a role^51^.

In this study we used an average score of subjective happiness and satisfaction with life as a measure of subjective well-being. Over the years several attempts have been undertaken to evaluate the specificity and overlap of the concepts that fall under the umbrella of SWB. Our findings contribute to this quest by showing that results are similar for happiness, satisfaction with life and the average score. This is in line with our earlier work where we show strong phenotypic and genetic correlations between happiness and satisfaction with life^4, 7, 52^.

In conclusion, this study is the first to reveal nonlinear relationships between subjective well-being and hippocampal volumes. The nonlinearities imply a reduction of SWB in subjects with relatively smaller hippocampal volumes and comparable levels of SWB in subjects with medium to higher hippocampal volumes. Stronger associations on the negative end of the brain-behavior spectrum are likely common and therefore, for future studies, we recommend to take into account the possibility of non-linear underlying functions. There were no significant, linear or quadratic, relations with volumes of the basal ganglia, thalamus, amygdala, and nucleus accumbens. The observed phenotypic relation between SWB and left and right hippocampal volumes is not explained by common genetic factors. Instead, other etiological factors, such as inadequate parental care, childhood trauma or later life exposure to stress with accompanying changes in stress hormone levels, may exert detrimental effects on both SWB and the hippocampus to bring about the observed association.

## Materials and Methods

### Subjects and sMRI

In total 724 registrants from the Netherlands Twin Register^53, 54^ had self-ratings of SWB and measurements of subcortical brain volumes. In 636 subjects of this total sample, DNA was also available. The total sample, summarized in Table 7, was composed of monozygotic (MZ) and dizygotic (DZ) twins and a number of additional siblings who had participated in 5 different MRI twin studies conducted by the Netherlands Twin Register and the University Medical Center Utrecht, The Netherlands. Using the unique registration numbers of our participants we ascertained that there were no replicated subjects among the 5 studies. All twins (and their parents in the case of children) provided written informed consent. The study and all methods, including the experimental protocols, were performed in accordance with relevant guidelines and regulations, and approved by the scientific committee of the VU University Amsterdam Faculty of Psychology and Education (MRI-study 1,2,3,5) and ethical review board of the VU medical center Amsterdam (MRI-study 1,2,3,5) and the Central Committee on Research involving Human Subjects of the Netherlands (CCMO: MRI-study 5) and scientific and ethical committee of the University Medical Center Utrecht (MRI-study 4,5).

**Table 7:** Description of sex distribution, SWB and age of SWB and MRI assessment.

MRI study	M/F	SWB	Age_SWB_	Range	Age_MRI_	Range	Correlation Age_SWB_-Age_MRI_
1	20/38	24.6 ± 3.7	18.1 ± 2.1	14.5–27.0	14.7 ± 1.5	11.0–18.0	0.59*
2	92/152	24.5 ± 4.4	31.3 ± 10.5	16.5–57.5	34.0 ± 10.2	19.0–57.0	0.98*
3	22/36	25.0 ± 4.4	32.6 ± 6.4	22.6–45.3	30.0 ± 5.9	20.0–42.0	0.94*
4	64/66	25.7 ± 4.1	37.6 ± 7.3	25.0–67.2	28.7 ± 6.5	19.1–55.9	0.93*
5	104/130	25.0 ± 3.7	17.2 ± 1.5	14.8–22.3	10.0 ± 1.3	9.0–15.0	0.91*
Total	302/422	24.9 ± 4.1	27.0 ± 10.8	14.5–67.2	23.4 ± 12.5	9.0–57.0	0.91*

MRI study: contributing twin MRI study; M/F: sex; SWB: mean (±SD) of SWB: Age_SWB_: mean (±SD) age of SWB assessment; Range: age range of SWB assessment; Age_MRI_: mean (±SD) age at time of MRI examination; Range: age range of MRI; correlation Age_SWB_-Age_MRI_: Pearson correlation between age at well-being assessment and age at MRI. *Statistically significant at the 0.01 level.

MRI-study 1^55^ was conducted by the Netherlands Twin Register and provided data from 26 complete MZ twin pairs, 3 single MZ twins and 1 DZ twin pair and 1 single DZ twin. The adolescent twins were selected to be concordant or discordant for ratings on the Child Behavior Checklist Attention Problem scale (CBCL-AP). MRI was obtained using a Siemens Sonata 1.5 Tesla scanner (Siemens, Erlangen, Germany) with a standard circularly polarized head coil. Three 3D MPRAGE T1-weighted sequence were run for all participants. Each volume consisted of 160 sagittal slices (1.00 × 1.00 × 1.00 mm), with an in plane voxel size of 1 mm^2^ (TR = 1900 ms; TI = 1100 ms; TE = 3.93 ms; flip angle = 15°; and 256 × 224 matrix).

Study 2^56^ was conducted by the Netherlands Twin Register and contributed data from 69 complete MZ twin pairs, 45 DZ pairs, 6 single MZ twins, 4 single DZ twins and 6 siblings. Selection criteria for this study included an age range between 18 and 60 years and discordant, or concordant high or low scores on the abbreviated version of the Padua Inventory Revised^57^ on obsessive-compulsive symptoms. Ten twins used antidepressant medication (6 used selective serotonin-reuptake inhibitors, 2 used selective serotonin and noradrenalin reuptake inhibitors, 1 used tricyclic antidepressants and 1 used Lithium). MRI was collected using a Philips Intera 3.0 Tesla scanner (Philips, Medical Systems, Best) with a standard SENSE receiver head coil. A 3D gradient-echo T1-weighted sequence (technique: T1TFE) was run for all participants. Each volume consisted of 182 coronal slices (1.00 × 1.00 × 1.20 mm), with an in-plane voxel size of 1 mm^2^ (TR = 9.64 ms; TE = 4.60 ms; flip angle = 8°; and 256 × 256 matrix).

Study 3^58^, conducted by the Netherlands Twin Register, contributed 24 complete MZ twin pairs, 2 DZ pairs, and 6 single MZ twins. Selection criteria included an age ranging between 18 and 50 years and discordant, or concordant anxious depression scores^59^. In this sample, 1 subject used antidepressant medication (selective serotonin-reuptake inhibitor) at the time of MRI scanning. A Siemens Sonata 1.5 Tesla scanner (Siemens, Erlangen, Germany) with a standard circularly polarized head coil was used. A 3D MP-RAGE T1-weighted sequence was run for all participants. Each volume consisted of 160 coronal slices (1.00 × 1.00 × 1.50 mm), with an in plane voxel size of 1 mm^2^ (TR = 15 ms; TI = 300 ms; TE = 7.00 ms; flip angle = 8°; and 256 × 176 matrix).

Study 4^60^ was conducted by the University Medical Center Utrecht, and contributed 15 complete MZ twin pairs, 30 DZ pairs, 8 single MZ twins, 11 single DZ twins, and 21 siblings. The subjects in this sample were phenotypically unselected adult twins, and their full siblings. MRI was collected using a Philips Gyroscan 1.5 Tesla scanner (Philips, Medical Systems, Best). A 3D spoiled gradient-echo T1-weighted sequence (technique: T1FFE) was run for all participants. Each volume consisted of 170–180 coronal slices (1.00 × 1.00 × 1.20 mm), with an in-plane voxel size of 1 mm^2^ (TR = 30 ms; TE = 4.60 ms; flip angle = 30°; and 256 × 256 matrix).

Study 5^61^ is an ongoing collaborative study by the Netherlands Twin Register and the University Medical Center Utrecht and contributed 36 complete MZ twin pairs, 45 complete DZ pairs, 1 triplet composed of an MZ male twin pair and a DZ female, 9 single DZ twins, and 60 of their siblings. This is a longitudinal study in which mentally healthy twins, and their siblings are assessed at three and eight year intervals. For the present study, we included brain MRI data from the initial baseline measurement consisting of 9-year-old MZ and DZ twins and an older sibling^62^. For MRI, a Philips Achieva 1.5 Tesla scanner was used at the UMCU. A three-dimensional T1-weighted coronal spoiled gradient echo scan of the whole head (160–180 contiguous slices; 1 × 1 × 1.2 mm; TR = 30 ms, TE = 4.6 ms, flip angle = 30°, voxels, field-of-view = 256 mm/70%, and 256 × 256 matrix).

General exclusion criteria for all subjects were history of brain damage or neurological illness, as assessed from self-report surveys, and contraindications for MRI (e.g., pregnancy, clips and devices in the body). As noted above, a very limited number of subjects (10 out of 244 subjects in study 2 and 1 of 58 subjects in study 3) used antidepressant medication. We did not exclude these participants, after confirming that leaving these subjects out of our analyses did not significantly change our results. Prior to processing, all scans were clinically evaluated by a trained radiologist to ensure the absence of radiological abnormalities of the brain. Subsequently, all scans were de-identified to ensure researcher blindness during image processing and analysis. For each subject, the intracranial volume (ICV) and left and right hemisphere volumes of the basal ganglia nuclei (caudate, putamen, pallidum), thalamus, hippocampus, amygdala and nucleus accumbens were measured using the FreeSurfer 5.1.0 structural imaging pipeline http://surfer.nmr.mgh.harvard.edu ^63, 64^.

### Well-being

Two measures of self-reported evaluations of subjective well-being (SWB) were used in the present study: (1) satisfaction with life (SAT) was assessed with the Satisfaction with Life Scale^65^. The scale consists of five items which had to be answered on a 7-point scale ranging from 1 = ‘strongly disagree’ to 7 = ‘strongly agree’. Example items are “My life is going more or less as I wished” and “I’m satisfied with my life”. Internal consistency of the scale was good with a Chronbach’s Alpha of 0.86. (2) Subjective happiness (HAP) was assessed with the Subjective Happiness Scale^66^. The scale consists of 4 items which had to be answered on 7-point scale ranging from 1 = ‘strongly disagree’ to 7 = ‘strongly agree’. Example items are “On the whole I’m a happy person” and “On the whole, I’m not very happy”. Internal consistency of the scale was good with a Chronbach’s Alpha of 0.84. Since, satisfaction with life and subjective happiness are highly correlated (0.73 for males and 0.77 for females) and load on one underlying genetic factor^4^, we computed the arithmetic mean score, across the individual’s scores on both scales, to represent participants subjective well-being (SWB). If longitudinal self-reports for satisfaction with life and/or subjective happiness were available the latest assessment was used to compute the mean SWB score.

Table 7 includes the mean age and age range (columns Age_SWB_ and Range) of the SWB measure per study, and the mean age and age range of the participants at the time of MRI (Age_MRI_ and Range). Pearson correlations between Age_SWB_ and Age_MRI_ (Correlation Age_SWB_-Age_MRI_) were high and statistically significant at the 0.01 level (Bonferroni 0.05/5 tests), indicating relatively consistent time intervals between SWB and MRI assessments. From the mean values for Age_SWB_ and Age_MRI_ in Table 7, one can see that the SWB assessment generally followed MRI with average delays ranging from 2.6 years (study 3) to 9 years (study 4). In study 2, however, SWB assessment preceded MRI, on average by 2.7 years.

### Phenotypic association tests

To test for associations of SWB with subcortical volumes, linear mixed models were run in IBM SPSS version 21.0 using maximum likelihood estimation. The dependent variable was the SWB score and variable of interest was the volume of the subcortical structure. To test for possible non-linear relations, we transformed the subcortical brain volumes to Z-scores, and included both a linear and quadratic volume term. Z-transformation was accomplished using the standard procedure as available in SPSS software, i.e., by using the “DESCRIPTIVES” command combined with the “/SAVE” option. This rescales the variables, to a mean of 0 and a standard deviation of 1, by subtracting a variable’s mean from each separate value and dividing the remainder by the variable’s standard deviation. The following variables were included as covariates: ICV (total Intra Cranial Volume), sex, and age at MRI acquisition. The variable MRI study (5 different MRI studies) was included as a factor. As the data were derived from family members (twins and siblings), we added genetic relatedness (1.0 between MZ twins and 0.5 between DZ twins and sibs) as a random effect to the models^67–69^. The chosen alpha level was 0.05/14 = 0.0036 (Bonferroni correction for 14 tests: 7 structures in the left and right hemisphere).

### Polygenic scores and Mendelian Randomisation

To investigate the role of underlying genetic predisposition we estimated polygenic scores for SWB and brain volume that indicate the correspondence of each subject with a set of genetic variants recently reported in large genome-wide association (GWA) studies on subjective well-being^7^ and subcortical brain volumes^31^. Polygenic scores were constructed using LDpred polygenic scores (LD-PGS) based on LDpred effect sizes^70^. LDpred adjust the effect sizes from the original meta-analysis (excluding the NTR cohort) for the effects of linkage disequilibrium (LD) using an external reference panel to estimate the LD structure among SNPs. As the LD reference panel, we used genotype data imputed to 1000 Genomes Phase 1 reference panel and converted to hard calls. Since HapMap3 SNPs are known to be most reliably imputed, the LD-PGS are based on HapMap3 SNPs only. We constructed all scores in PLINK^71^ using allelic dosages of genotypes imputed to 1000G Phase 1. The fraction of causal SNPs was set to 1. In total 1,059,064 SNPs were used to construct the scores. Genotyping was done on a series of arrays and imputed genotype scores.

Causal directions in observed SWB-brain relations were investigated by means of Mendelian Randomization using a 2 stage least squares (2SLS) approach^30^. To test if SWB variation induced by genes could have influenced brain volume we first computed the variance in SWB explained by the polygenic scores for SWB. In the second step we then tested for association between the explained SWB variance and the measured brain volume(s). Conversely, to test for the possibility that brain volume variation induced by genes could have influenced SWB we computed the variance in brain volume explained by the polygenic score for brain volume and then tested for association between the explained brain volume variance and individual SWB scores. For the regression steps of the 2SLS procedure we used linear mixed modeling with random effects to accommodate shared family backgrounds. To account for nonlinear associations between SWB and brain volume, we additionally divided the residual “environmental” variance from the first step of the 2SLS procedure into three equal sized strata and included this variable (coded 1 to 3) as an interaction term in the second step of the 2SLS procedure^72^. In addition, in both steps of the 2SLS method, we included sex, age at MRI assessment, and MRI study (coded 1 to 5) as covariates. Furthermore, we added 3 principal components to correct for Dutch population substructures, 1 principal component to account for batch effects and 7 principal components to account for different genotyping platforms. For an extensive description of how the principal components were computed, see Abdellaoui *et al*.^73^. Finally, we excluded subjects with known non-European ancestry. These individuals were identified by projecting PCs from the 1000 Genomes individuals on the Dutch individuals, and with additional help of the birth country of their parents (available for 60% of the subjects). Eight of the top ten 1000 Genomes PCs (all but PC4 and PC7) cluster the European populations together, making them useful for detecting individuals with a non-European ancestry. A Dutch individual was labeled as a potential outlier with a non-European ancestry if one of the 1000 Genomes PCs of that individual was lower than the minimum or higher than the maximum score of that particular PC of the European 1000 Genomes individuals (CEPH, Finnish, British, Iberian, and Toscan).

### Twin Modeling

For significant SWB-brain relations we estimated the 5 × 5 genetic and environmental covariance matrices of SWB, the subcortical volumes, and the subcortical volumes squared using covariance structure modeling of monozygotic (MZ) and dizygotic (DZ) twin data. We included study, age and sex as fixed covariates. Letting Σ_A_, Σ_D_, and Σ_E_ denote the 5 × 5 additive genetic (A), dominance genetic (D), and unshared environmental (E) covariance matrices. The 5 × 5 phenotypic covariance matrix equals Σ_Ph_ = Σ_A_ + Σ_D_ + Σ_E_. The expected 10 × 10 covariance MZ and DZ twin covariance matrices are:

1 $$\begin{array}{lll}{{\rm{\Sigma }}}_{{\rm{MZ}}} & = & {{\rm{\Sigma }}}_{{\rm{A}}}+{{\rm{\Sigma }}}_{{\rm{D}}}+{{\rm{\Sigma }}}_{{\rm{E}}}{{\rm{\Sigma }}}_{{\rm{A}}}+{{\rm{\Sigma }}}_{{\rm{D}}}\\  &  & {{\rm{\Sigma }}}_{{\rm{A}}}+{{\rm{\Sigma }}}_{{\rm{D}}}{{\rm{\Sigma }}}_{{\rm{A}}}+{{\rm{\Sigma }}}_{{\rm{D}}}+{{\rm{\Sigma }}}_{{\rm{E}}}\end{array}$$

2 $$\begin{array}{ccc}{{\rm{\Sigma }}}_{{\rm{DZ}}} & = & {{\rm{\Sigma }}}_{{\rm{A}}}+{{\rm{\Sigma }}}_{{\rm{D}}}+{{\rm{\Sigma }}}_{{\rm{E}}}{\rm{0.5}}* {{\rm{\Sigma }}}_{{\rm{A}}}+0.25* {{\rm{\Sigma }}}_{{\rm{D}}}\\  &  & {\rm{0.5}}* {{\rm{\Sigma }}}_{{\rm{A}}}+{\rm{0.25}}* {{\rm{\Sigma }}}_{{\rm{D}}}{{\rm{\Sigma }}}_{{\rm{A}}}+{{\rm{\Sigma }}}_{{\rm{D}}}+{{\rm{\Sigma }}}_{{\rm{E}}}\end{array}$$

In equation 2, the 0.5 and 0.25 are the expected additive genetic and dominance correlations, respectively, among DZ twins. The correlations both equal 1.0 in the case of MZ twins, because MZ twins are genetically identical.

Using raw data maximum likelihood (ML) estimation in OpenMx^74^, we first estimated the unconstrained phenotypic 10 × 10 covariance MZ and DZ matrices, to obtain ML estimates of these covariance matrices, and a baseline log likelihood. We then estimated these covariance matrices subject to the ADE constraints, as shown above. Subsequently we used likelihood ratio tests (a.k.a., chi-square difference tests) to 1) evaluate the fit of the ADE model, 2) to test whether Σ_D_ can be dropped, and 3) to test the genetic and environmental correlations between SWB and subcortical brain volume(s).

### Genetic Correlation (rg) from published GWAS

Finally, we also determined the genetic correlation between SWB and subcortical brain volume(s) by comparing the GWAS summary statistics reported for SWB^7^ and subcortical brain volume^31^. The genetic correlation (rg) was computed using bivariate LD score regression^34, 35^ with reference LD scores based on genotypic data from a European-ancestry population^75^.

### Data Availability

Data come from the Netherlands Twin Register (NTR). Due to privacy of the participants, these data are accessible after a data request application to the NTR and University Medical Center Utrecht (for MRI data from studies 4 and 5).
