# Publisher Correction: Associations between subjective well-being and subcortical brain volumes

**DOI:** 10.1038/s41598-018-35961-9

**Published:** 2018-11-28

**Authors:** D. van ’t Ent, A. den Braber, B. M. L. Baselmans, R. M. Brouwer, C. V. Dolan, H. Hulshoff Pol, E. J. C. de Geus, M. Bartels

**Affiliations:** 1 Department of Biological Psychology, VU University, Amsterdam, The Netherlands; 2 EMGO+ Institute for Health and Care Research, VU University Medical Center, Amsterdam, The Netherlands; 3 Amsterdam Neuroscience, Amsterdam, The Netherlands; 4 Alzheimer Center and Department of Neurology, VU University Medical Center, Amsterdam, The Netherlands; 5 Brain Center Rudolf Magnus, Department of Psychiatry, University Medical Center Utrecht, Utrecht, The Netherlands

Correction to: *Scientific Reports* 10.1038/s41598-017-07120-z, published online 31 July 2017

This Article contains typographical errors in Equation (1), where

$$\begin{array}{rcl}{{\rm{\Sigma }}}_{{\rm{MZ}}} & = & {{\rm{\Sigma }}}_{{\rm{A}}}+{{\rm{\Sigma }}}_{{\rm{D}}}+{{\rm{\Sigma }}}_{{\rm{E}}}\,{{\rm{\Sigma }}}_{{\rm{A}}}+{{\rm{\Sigma }}}_{{\rm{D}}}\\  &  & {{\rm{\Sigma }}}_{{\rm{A}}}+{{\rm{\Sigma }}}_{{\rm{D}}}\,{{\rm{\Sigma }}}_{{\rm{A}}}+{{\rm{\Sigma }}}_{{\rm{D}}}+{{\rm{\Sigma }}}_{{\rm{E}}}\end{array}$$

should read:

$$\begin{array}{rcll}{{\rm{\Sigma }}}_{{\rm{MZ}}} & = & {{\rm{\Sigma }}}_{{\rm{A}}}+{{\rm{\Sigma }}}_{{\rm{D}}}+{{\rm{\Sigma }}}_{{\rm{E}}} & {{\rm{\Sigma }}}_{{\rm{A}}}+{{\rm{\Sigma }}}_{{\rm{D}}}\\  &  & {{\rm{\Sigma }}}_{{\rm{A}}}+{{\rm{\Sigma }}}_{{\rm{D}}} & {{\rm{\Sigma }}}_{{\rm{A}}}+{{\rm{\Sigma }}}_{{\rm{D}}}+{{\rm{\Sigma }}}_{{\rm{E}}}\end{array}$$

In addition, Equation (2) contains typographical errors, where

$$\begin{array}{rcl}{{\rm{\Sigma }}}_{{\rm{DZ}}} & = & {{\rm{\Sigma }}}_{{\rm{A}}}+{{\rm{\Sigma }}}_{{\rm{D}}}+{{\rm{\Sigma }}}_{{\rm{E}}}{\rm{0.5}}\ast {{\rm{\Sigma }}}_{{\rm{A}}}+{\rm{0.25}}\ast {{\rm{\Sigma }}}_{{\rm{D}}}\\  &  & {\rm{0.5}}\ast {{\rm{\Sigma }}}_{{\rm{A}}}+{\rm{0.25}}\ast {{\rm{\Sigma }}}_{{\rm{D}}}\,{{\rm{\Sigma }}}_{{\rm{A}}}{+{\rm{\Sigma }}}_{{\rm{D}}}{+{\rm{\Sigma }}}_{{\rm{E}}}\end{array}$$

should read:

$$\begin{array}{rcll}{{\rm{\Sigma }}}_{{\rm{DZ}}} & = & {{\rm{\Sigma }}}_{{\rm{A}}}+{{\rm{\Sigma }}}_{{\rm{D}}}+{{\rm{\Sigma }}}_{{\rm{E}}} & \,{\rm{0.5}}\ast {{\rm{\Sigma }}}_{{\rm{A}}}+{\rm{0.25}}\ast {{\rm{\Sigma }}}_{{\rm{D}}}\\  &  & {\rm{0.5}}\ast {{\rm{\Sigma }}}_{{\rm{A}}}+{\rm{0.25}}\ast {{\rm{\Sigma }}}_{{\rm{D}}} & {{\rm{\Sigma }}}_{{\rm{A}}}+{{\rm{\Sigma }}}_{{\rm{D}}}+{{\rm{\Sigma }}}_{{\rm{E}}}\end{array}$$

